# Cortical modulation of BOLD signals in white matter

**DOI:** 10.21203/rs.3.rs-5931986/v1

**Published:** 2025-02-03

**Authors:** Zhaohua Ding, Lyuan Xu, Yurui Gao, Yu Zhao, Yicheng Tan, Adam W. Anderson, Muwei Li, John C. Gore

**Affiliations:** 1 Vanderbilt University Institute of Imaging Science, Vanderbilt University Medical Center; Nashville, TN, USA 37232; 2 Department of Electrical and Computer Engineering, Vanderbilt University; Nashville, TN, USA 37232; 3 Department of Biomedical Engineering, Vanderbilt University; Nashville, TN, USA 37232; 4 Department of Computer Science, Vanderbilt University; Nashville, TN, USA 37232; 5 Department of Radiology, and Functional and Molecular Imaging key Laboratory of Sichuan Province, West China Hospital of Sichuan University; Chengdu, China 610041; 6 Huaxi MR Research Center, West China Hospital of Sichuan University; Chengdu, China 610041; 7 Research Unit of Psychoradiology, Chinese Academy of Medical Sciences; Chengdu, China 610041; 8 School of Electronic Engineering, Xidian University; Xi’an, China 710126; 9 Department of Radiology and Radiological Sciences, Vanderbilt University Medical Center; Nashville, TN, USA 37232; 10 Department of Physics and Astronomy, Vanderbilt University; Nashville, TN, USA 37232; 11 Molecular Physiology and Biophysics, Vanderbilt University; Nashville, TN, USA 37232

## Abstract

The relationship of BOLD signals in white matter to cortical neural activity remains unclear. We quantified the degree to which spontaneous neural activities in the cortex, which are reflected in low frequency fluctuations in cortical BOLD signals, modulate BOLD signals in white matter. From measurements of resting state correlations we find cortical networks of more basic level functions tend to contribute more to correlated fluctuations in white matter than those of higher level functions. In addition, each cortical network exhibits distinct, structurally interpretable spatial distribution patterns of white matter projections. Moreover, the myelination level of cortical networks is found to be strongly correlated with the white matter projection of cortical BOLD signals. Our findings confirm that BOLD signals in white matter encode neural activity in proportion to the spontaneous activity of individual cortical networks, and with network-specific spatial distribution patterns, which could be mediated by the microstructure of the brain cortex.

## Introduction

Magnetic resonance imaging (MRI) based on blood-oxygenation-level-dependent (BOLD) signals has become a primary functional neuroimaging technique that is widely used by the neuroscience community. BOLD functional MRI (fMRI) has been used for localizing cortical activities^1^ and mapping their inter-regional correlations^2^, but the significance of BOLD effects in white matter has not been recognized to a similar extent^3,4^. However, there is evidence that metrics of neural activity are encoded in white matter BOLD signals^5–8^, which is further supported by white matter structural organization^9^, glucose metabolism^10^, electrophysiology^11^, and transcriptomic profiling^12^. However, concerns persist that the BOLD signal changes in white matter could be driven by physiological or experimental confounds that are not of neural origin^3^.

In this note, we address some of these concerns by establishing relationships between neural activities within the cortex at rest and BOLD signals measurable in white matter. Previous reports have demonstrated that the cortex exhibits regionally-varying levels of neural activity with different temporal profiles^13^, and that neural signals from each region traverse the white matter through a cascade of cortical responses due to the network architecture of the human brain^14,15^. Given these findings, we hypothesize that cortical networks showing higher neural activity modulate signals in white matter more strongly, with each network imposing distinct patterns in white matter. Here we evaluate this hypothesis and also quantify the myelin content in different cortical networks in order to derive insight into the relationships between cortical activities and white matter BOLD signals.

## Results

**Figure 1.**
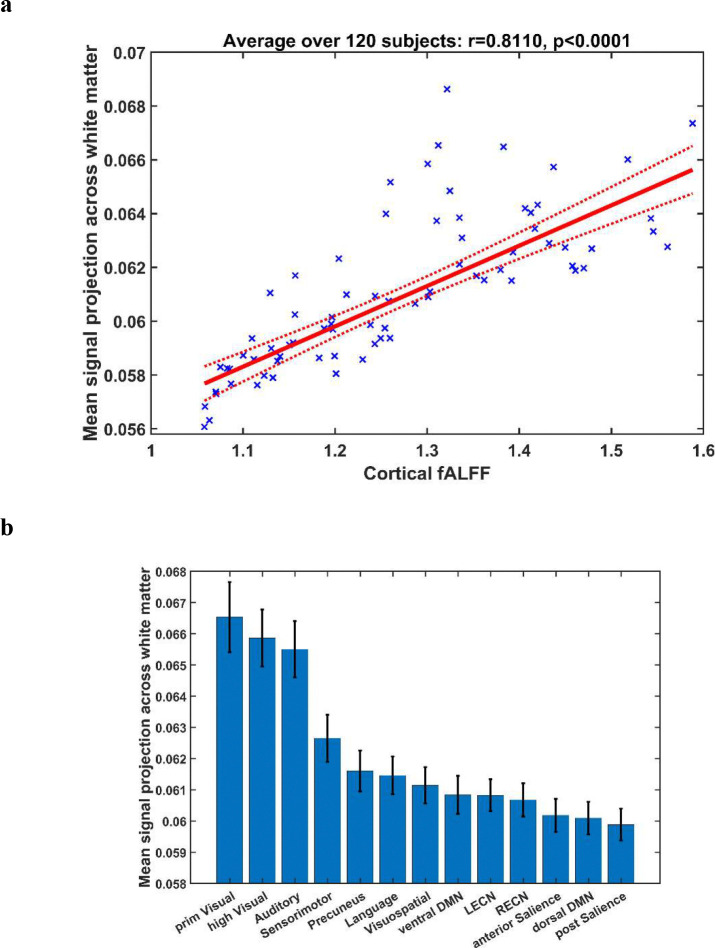
(a) Relationship between subject-averaged fALFF of cortical BOLD signals and their subject-averaged mean white matter projection. Each data point represents subject-averaged measures for an ROI in the cortex. (b) Mean white matter projection of BOLD signals in the cortical functional networks analyzed. The vertical line at the top of each bar represents standard error across the 120 subjects studied. Abbreviations: prim = primary, DMN = default mode network. LECN = left executive control network. RECN = right executive control network.

We analyzed functional images of 120 healthy young adults sourced from publicly available data. For each subject, the cortical gray matter was parcellated into 75 regions of interest (ROIs), and the fractional amplitude of low-frequency fluctuations (fALFF) of BOLD signals was computed for each ROI (see Methods for details). Figure 1(a) shows the relationship between the fALFF of cortical BOLD signals and their mean white matter projections (i.e., mean value across white matter projection map). For the 75 ROIs studied, the mean white matter projection averaged over the 120 subjects studied was strongly correlated (r=0.8110, p<0.0001) with the subject-averaged fALFF (see Supplementary Fig. S1 in Supplementary Information for correlation results from each individual subject). To examine whether the observed correlations were spurious, we randomly perturbed the time order of BOLD signals in white matter and repeating the above analysis with 60 subjects, which yielded a mean r=−0.1967±0.2665 (see Supplementary Fig. S2 in Supplementary Information for detailed results).

In our control experiment using skull bone marrow, we excluded BOLD datasets with visible image distortion or insufficient thickness of bone marrow in the skull, and thus ended up with 24 subjects with robustly defined skull bone marrow (see Supplementary Fig. 3a-e for a typical example of bone marrow voxel locations). Applying the same procedure as for white matter (without correction for time delay) showed that the correlation coefficient between fALFF of cortical BOLD signals and their mean bone marrow projection was r=0 (see Supplementary Fig. 3f). On the individual subject level, the mean correlation coefficient was r=0.1310±0.2445.

Our detailed regional analysis reveals that BOLD signals in the 13 cortical functional networks had varying levels of white matter projection, and overall, those of more basic level functions (auditory, primary/higher visual, and sensorimotor) had greater signal projection in white matter than those of higher level functions (see Fig. 1(b)).

**Figure 2.**
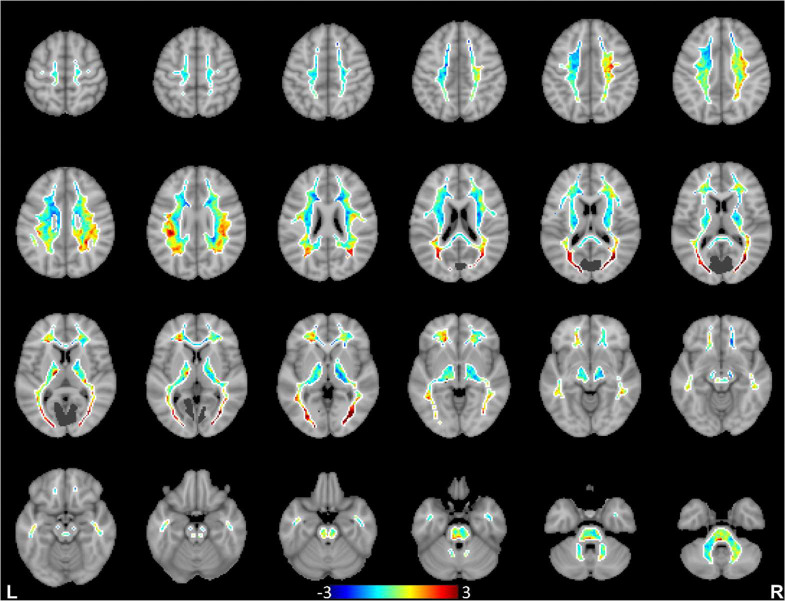
Spatial distributions of white matter projections of BOLD signals from the primary visual network. Data are averaged across the 120 subjects, and dark gray color denotes cortical regions of the primary visual network.

**Figure 3.**
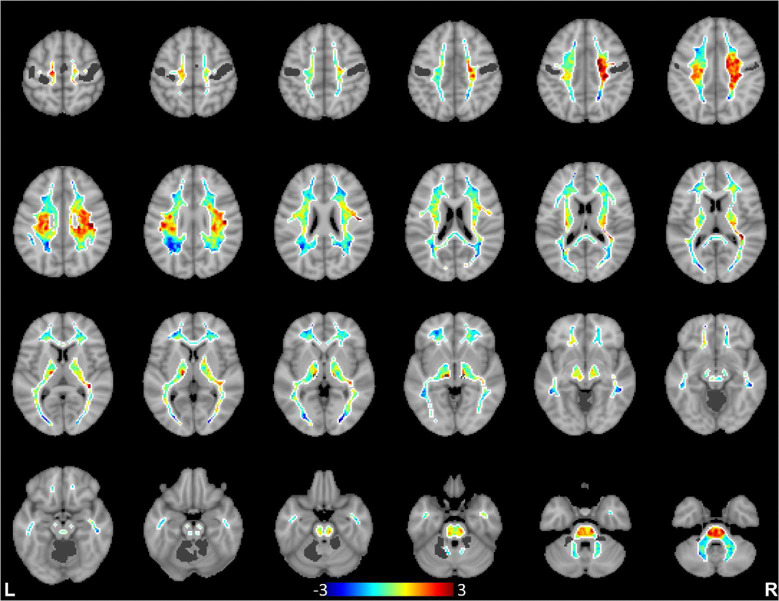
Spatial distributions of white matter projections of BOLD signals from the sensorimotor network. Data are averaged across the 120 subjects, and dark gray color denotes cortical regions of the sensorimotor network.

**Figure 4.**
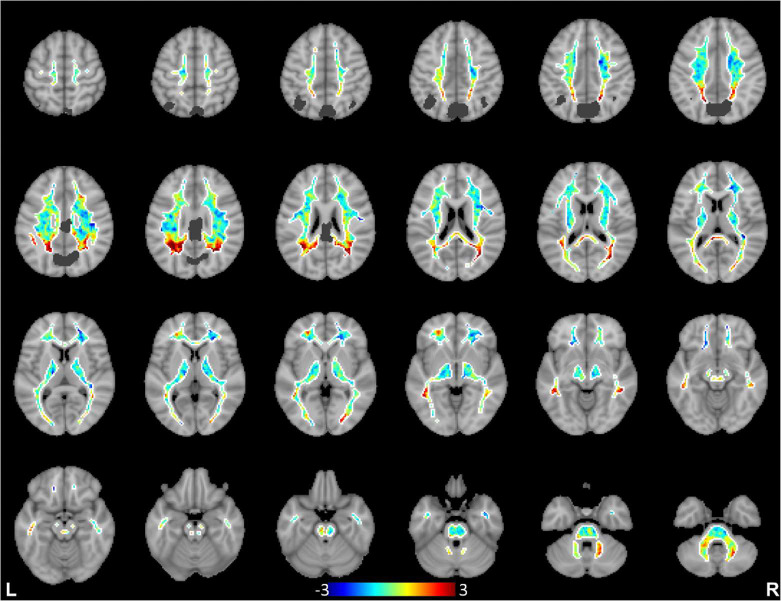
Spatial distributions of white matter projections of BOLD signals from the precuneus network. Data are averaged across the 120 subjects, and dark gray color denotes cortical regions of the precuneus network.

We further computed spatial distributions of BOLD signal projections in white matter for all the cortical functional networks. Each network produced a distinct spatial pattern in white matter, with high projection regions generally close to the network cortices. Remote white matter projections were also commonly seen, indicating BOLD effects transferred through white matter to remote cortices within the network or to other related networks. To illustrate the distinctness of the spatial distribution patterns of individual networks, the distribution maps of white matter projections of the primary visual, sensorimotor and precuneus networks are compared in Figs. 2, 3&4 (see Supplementary Fig. S5 in Supplementary Information for distribution maps of all the 13 cortical networks studied). It can be appreciated that these cortical networks tended to have greater signal projection respectively along the optic radiations (primary visual), along the projection pathways (sensorimotor), and in the parietal lobe (precuneus).

**Figure 5.**
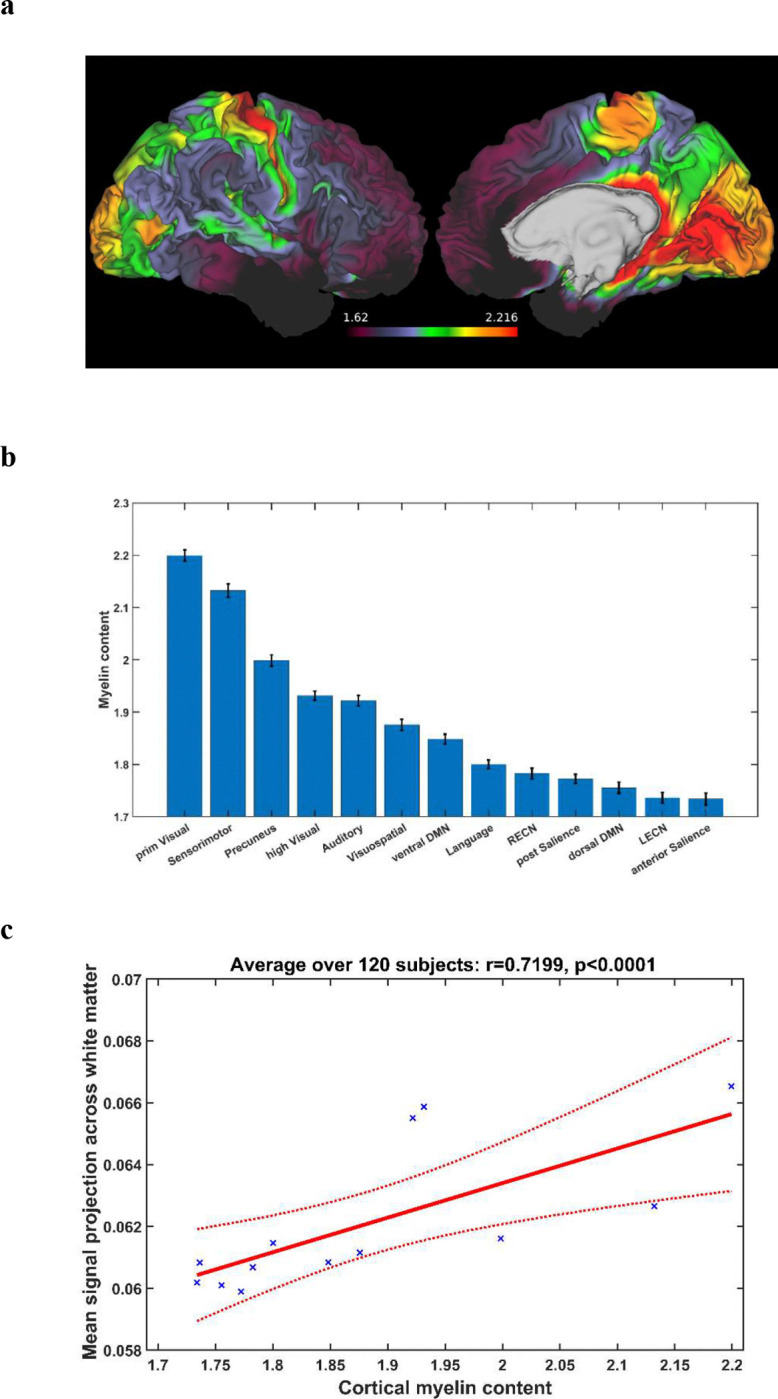
(a) Mid-thickness cortical surface map of mean myelin content across the 120 subjects studied. Left: lateral surface of the right hemisphere. Right: medial surface of the right hemisphere. (b) Mean myelin content in the cortical functional networks analyzed. The vertical line at the top of each bar represents standard error across the 120 subjects studied. Abbreviations: prim = primary, DMN = default mode network. LECN = left executive control network. RECN = right executive control network. (c) Relationship between myelin content and mean white matter projection of BOLD signals in the cortical functional networks analyzed. Each data point represents the subject-averaged measures for a cortical functional network.

The myelin content across the mid-thickness cortical surface is graphically rendered in Fig. 5(a). In keeping with several other reports^16,17^, the cortical regions of heavier myelination tended to concentrate in the occipital, temporal and parietal lobe, where the primary visual, higher visual, auditory and sensorimotor networks reside. Fig. 5(b) shows the mean myelin content for the 13 cortical networks analyzed. Networks engaged in more basic functions tended to have heavier myelination than those of higher level functions. The cortical myelin content is correlated against the mean white matter projection of cortical BOLD signals in Fig. 5(c). There was a strong positive correlation between the two measures (r=0.7199, p<0.0001), suggesting that the cortical networks of heavier myelination tended to impart greater signals to white matter.

## Discussion

We calculated the cross-correlations between voxels in defined cortical ROIs and all voxels in white matter, and averaged their values across ROIs engaged in specific networks to quantify the influence of cortical activity on white matter BOLD signals in a resting state. Detailed regional analysis shows that different cortical networks modulate white matter BOLD signals differently, with each network exhibiting distinct, structurally interpretable, spatial distribution patterns of signal contributions. Quantitatively, the cortical networks of more basic functions tend to exert stronger influence on BOLD signals in white matter than those of higher level functions. This spectrum of activity agrees well with cortical myelin distributions observed in this study, and with previous reports on cortical structural connectivity profiles^18^, functional connectivity gradients^19,20^, cortical microstructure gradients^21^, and evolutionary explanation patterns^22^.

It has been well-established that low frequency fluctuations in cortical BOLD signals are closely related to spontaneous neural activity^2^. Our findings from this study confirm that fluctuations in white matter BOLD signals encode neural activity similarly to gray matter. The observation that components of white matter BOLD signals are closely associated with spontaneous activities of individual cortical networks, which exhibit network-specific spatial distribution patterns, can be most reasonably explained as being driven by neural activities instead of physiological artifacts. However, there is a concern of signal contaminations from nearby gray matter, which could arise from limited imaging resolution, imperfect image registration, spatial smoothing and other technical issues. This study performed a voxel-wise analysis with conservative white matter masking which should largely ameliorate this concern. In addition, effects of progressively tighter white matter masking were also examined during the study, which showed that the relationship between fALFF and mean white matter projection of cortical BOLD signals continued to hold well. In particular, when white matter was masked to contain only a small deep region with a total of 57 voxels, where signal contaminations from gray matter could be factually ignored, the correlation as we observed in Fig. 1(a) was still as high as r = 0.7108 (see Supplementary Fig. S6 in Supplementary Information for detail), although presumably the signal-to-noise ratio in this small deep white matter region was much lower.

Another concern is that the relationship we observed between BOLD signals in the cortex and white matter might be mediated by global signal across the brain rather than by network-specific neural signals. The issue of global signal indeed has been quite perplexing as inferences regarding brain connectivity profiles are sensitive to whether or not the global signal is regressed out^23,24^. Recognizing that global signal computed from brain parenchyma may contain neural information^25^, we chose to regress out the mean cerebrospinal fluid signal (CSF) in this work with the expectation that the effect of global signal is reduced with a minimal loss of neural information^7,26^. Notwithstanding the regression of CSF signal (along with head movements, cardiac and respiratory signals and linear temporal trends), it is still possible that residual global signal might have created artificial correlations between the cortex and white matter. To examine this possibility, we used skull bone marrow as a control tissue for white matter and correlated signal fluctuations in the bone marrow against cortical fALFF, which yielded zero correlation coefficient (r=0, p=0.7282). In addition, we correlated the projection of cortical BOLD signals in white matter against cortical fALFF without timing correction and found that, on average, the correlation coefficient was reduced ~6% . Mechanistically, the effect of global signal would have been more pronounced without any timing corrections to a particular tissue type. Therefore, this finding, along with that from the bone marrow experiment, have basically ruled out that the global signal was a major contributor to the correlation patterns observed in this work.

Finally, there is a possibility that the fluctuations in white matter BOLD signals might be driven by effects of vessels draining from upper stream gray matter. This is however unlikely since gray matter and white matter have two distinct venous systems^27^ and arterial supplies^28^ that have no mutual spatial overlap. Moreover, an earlier finding that the brain parenchyma has quite uniform oxygen extraction fraction is indeed a counter-argument against the vessel draining speculation^29^. Nonetheless, to fully eliminate the possibility of vessel draining effects, additional in vivo human experiments are needed but clearly justified.

## Methods

### Image Data and Study Subjects

This study used publicly available data (the Human Connectome Project database^30^), from which images of 120 young adults (female = 60, age range = 26–35 years) were randomly selected. The resting-state fMRI data used in this study were acquired with multiband gradient-echo echo-planar imaging sequences with the following parameters: repetition time (TR) = 720 ms, echo time = 33.1 ms, voxel size = 2×2×2 mm^3^, number of volumes = 1200. The fMRI data were minimally preprocessed^31^, and were further processed in this study to regress out nuisance variables from head movements, cardiac and respiratory signals^32^, and the mean CSF signal, followed by removal of linear temporal trends.

### Image Data Analysis

#### Power spectra of cortical BOLD signals:

The gray matter of each study subject was parcellated into 85 functional ROIs using an atlas defined previously^33^. These ROIs were confined to a common gray matter mask created using the MNI152 template. Note that the original atlas contains 90 gray matter ROIs, but five of them that reside in the basal ganglia network were excluded from our analysis. Also, ten cortical ROIs had insufficient number of voxels in the region (<7) and thus were further excluded so that only 75 cortical ROIs entered our subsequent analysis. Second, the power spectra of BOLD signals were computed for each of the cortical ROIs using Welch’s method^34^. Briefly, the BOLD time series from each voxel was segmented into eight consecutive and overlapping blocks, from which a periodogram was derived for each block and these were then averaged across the blocks. From the averaged periodogram, the fALFF was calculated as the ratio of average power across the frequency range of 0.01–0.08 Hz to the average power across all frequencies above 0.01 Hz. Finally, the resulting fALFF values were averaged across each of the cortical ROIs.

#### Projections of cortical BOLD signals onto white matter:

A common white matter mask was created using the MNI152 template with a tight threshold of 0.8. To avoid signal contaminations from nearby gray matter, the most superficial white matter was eroded by using a structuring element of six directly adjacent neighbors. BOLD signals in the eroded white matter mask were then slightly smoothed with an FWHM=2 mm Gaussian kernel, bandpass filtered to retain signals in the frequency range from 0.01 to 0.15 Hz, and normalized into unit variance. Meanwhile, cortical BOLD signals were similarly bandpass filtered and normalized.

For each voxel in the cortical ROIs, the product of its BOLD time series and that in a white matter voxel was computed and integrated to represent the projection of the cortical BOLD time series onto the white matter voxel. This projection or inner product is the same as the cross-correlation at zero lag. To take into account the time delay in white matter BOLD signals^7^, progressive delays of 1–10 TR were added in moving from superficial to deep white matter prior to computations of the signal projections. This process yielded a (time delay corrected) white matter projection map for each voxel in the cortical ROIs. The average of the white matter projection maps for all voxels in a cortical ROI was defined to be the white matter projection map of the ROI.

#### Projections of cortical BOLD signals onto skull bone marrow:

As a control experiment for white mater, a portion of bone marrow in the skull was analyzed using the same procedure as above. Specifically, a cuboid region that contained 5×16×5 voxels was first defined in each side of the head in temporally averaged BOLD data. These voxels were thresholded such that each cuboid retained 60 voxels of high intensity value, which were used as bone marrow control voxels. BOLD time series from the cortical ROIs were then projected onto these control voxels.

#### Network-based analysis:

The 75 cortical ROIs were grouped into 13 functional networks^33^, which contained basic functional networks (such as auditory and visual circuits) as well as cognitive networks (such as language and default mode network). A projection map was derived for each of these networks by averaging the white matter projection maps of all the cortical ROIs within the network, which was rescaled by voxel-wise normalization of the summed projection across the 13 networks into unity.

#### Quantification of cortical myelin content:

Cortical myelin maps of the selected subjects were sourced from the same HCP database as the fMRI images, and the mean myelin content for each of the 13 functional networks in each subject was calculated by averaging across the network.

## Data Availability

Processed data are available through request to the corresponding author Zhaohua Ding. Data in the supplementary information will be available online after acceptance for publication.
